# Primary spinal intradural mesenchymal chondrosarcoma with detection of fusion gene HEY1-NCOA2: A paediatric case report and review of the literature

**DOI:** 10.3892/ol.2014.2364

**Published:** 2014-07-18

**Authors:** CAROLA ANDERSSON, GUSTAF ÖSTERLUNDH, FREDRIK ENLUND, LARS-GUNNAR KINDBLOM, MAGNUS HANSSON

**Affiliations:** 1 Department of Clinical Pathology and Cytology, Sahlgrenska University Hospital, Gothenburg SE-413 45, Sweden; 2 Department of Pediatrics, The Queen Silvia Children’s Hospital, University of Gothenburg, Gothenburg SE-416 85, Sweden; 3 Department of Musculoskeletal Pathology, Royal Orthopedic Hospital, NHS Foundation Trust, Birmingham B31 2AP, UK; 4 Department of Medical Biosciences, Section for Pathology, Umeå University, SE-901 85 Umeå, Sweden

**Keywords:** chondrosarcoma, bone tumour, intradural, HEY1-NCOA2, fusion gene, sarcoma

## Abstract

Mesenchymal chondrosarcoma is an extremely rare malignant tumour that most commonly originates in the bone, but is also present in extraskeletal sites. The tumour is morphologically characterized by a biphasic pattern of small round cells and islands of cartilage. Spinal mesenchymal chondrosarcomas are even rarer and, therefore, few investigations exist regarding the biological behaviour of the tumours. In the present study, we report a case of a 10-year-old female presenting with 9 months of back pain and radiographic findings of an intradural lesion measuring 1.5 cm at the level of Th4. The tumour was completely excised and subjected to pathological analyses. Following detection of the HEY1-NCOA2 fusion gene, the tumour was morphologically and immunohistochemically defined as an intradural mesenchymal chondrosarcoma attached to the dura mater. In this study, we validate the recent identification of the fusion gene HEY1-NCOA2 in paediatric extraskeletal mesenchymal chondrosarcomas. The relevant literature is reviewed and further discussed in relation to our findings.

## Introduction

Mesenchymal chondrosarcoma (MCS), initially described by Lichtenstein and Bernstein in 1959 (1), is one of the most unusual chondrosarcomas, representing only 2–10% (2–5) of these tumour types worldwide. MCS arises across a broad age spectrum, generally between 20 and 40 years, but has also been diagnosed in the paediatric population globally. This chondrosarcoma type has been characterized as a high-grade tumour with a propensity to metastasise to the lung, lymph nodes and bone (6,7). MCS most frequently originates in the bone, but is located in soft tissues in ~25% of cases, and is occasionally detected adjacent to meninges and within the spinal canal (8). Until recently, MCS has lacked a specific diagnostic immunohistochemical profile or consistent genetic alterations that facilitate its differentiation from other bone tumours, and the diagnosis is generally based on histological features, which vary considerably. The current treatment of choice for MCS is surgery. To date, the efficacies of adjuvant chemo- and radiotherapy remain poorly defined (9), but appear to improve clinical outcomes. However, prognosis is extremely variable, as reflected in the published 10-year overall survival rates, ranging from 21% (10) to 67% (9). Improved understanding of the cell biology of MCS would therefore present a major advantage in accelerating the development of targeted drugs with enhanced effectiveness for tumour treatment. Limited information is currently available regarding the biology of MCS, with recorded cases of intraspinal MCS being extremely rare.

Tumour-specific, balanced chromosomal translocations have been identified in several histologically defined soft tissue sarcomas over the last 20 years (11,12). The first of these translocations was discovered in Ewing’s sarcoma (13,14), and subsequent reports have frequently demonstrated the specificity of these fusion genes (15,16).

No consistent molecular markers have been established for MCS until recently, although chromosomal reciprocal translocations have been reported, such as (11;22)(q24;q12) (17) and genetic findings of trisomy 8 (17,18). In early 2012, a novel fusion gene, HEY1-NCOA2, was identified in MCS (19). In the current study, we further confirmed the presence of the recently identified HEY1-NCOA2 fusion in a paediatric case of primary intradural MCS, supporting its utility as a novel diagnostic marker for the disease. The study was approved by the Regional Ethics Committee (Ethical Review Boards; Gothenburg, Sweden) of the CWS Soft Tissue Tumour Registry and written informed consent was obtained from the patient’s parents.

## Case report

### Case report

A 10-year-old female presented to the Södra Älvsborg Hospital (Borås, Sweden) with 9 months of back pain. General physical examination revealed normal results. The patient was referred to the Queen Silvia Children’s Hospital (Gothenburg, Sweden) and the data from neurological tests, including mental status, cranial nerve examination, cerebellar testing, and motor and sensory tests of the lower extremities, were additionally normal. No clinical evidence of a tumour was identified and the patient had no family history of cancer or genetic disorders. Magnetic resonance imaging (MRI) disclosed a 1.5-cm solid intradural lesion at the level of Th4. Further laboratory tests showed no abnormalities. The patient underwent surgery, with macroscopically complete removal of a well-defined tumour attached to the arachnoid roots, but not the dura mater or medulla spinalis. Following recovery from surgery, the patient was subjected to radiotherapy (proton radiation), specifically, 50.4 Gy in 1.8-Gy fractions over a period of 6 weeks. The patient is currently free of symptoms at two years following the completion of therapy. No radiological findings of relapse have been detected following MRI every four months.

### Histological and immunohistochemical analysis

All laboratory work, including morphological, molecular pathological and immunohistochemical analysis, was performed at the time of diagnosis. Resected tumour tissue was fixed in 10% formalin, cut into small pieces and embedded in paraffin. Tissue blocks were cut into 4-μm thick slices and stained with haematoxylin and eosin.

For immunohistochemical analysis, the following monoclonal primary antibodies were used: monoclonal rabbit anti-human CD99 (Epitomics, Burlingame, CA, USA) diluted 1:1000, monoclonal mouse anti-human Ki-67 [Flex Ready-to-Use (RTU) IR626; Dako, Carpinteria, CA, USA] and monoclonal rabbit anti-human S-100 (Flex RTU IR504; Dako). Positive controls (S-100 for the wall of appendix vermiformis; and CD99 and Ki-67 for the tonsils) were used for all staining protocols, while the negative controls were without primary antibodies. Slides were automatically stained using a Dako Autostainer (LV-1 Autostainer; Dako). For CD99 staining, slides were rehydrated with xylene, followed by a series of alcohol dilutions. Antigen retrieval was performed using Tris-EDTA (pH 9.0) in combination with heat induction in a microwave oven (8 min at 750 W, followed by 15 min at 350 W). Regarding Ki-67 and S-100 staining, the following procedure was used: PT Link (Pre-Treatment Module for Tissue Specimens; Dako) for Ki-67 Target Retrieval Solution, Low pH (K8005; Dako) and for S-100 Target Retrieval Solution, High pH (K8004; Dako). Slides were rinsed twice in buffer (EnVision™ FLEX Buffer; 8007; Dako), with subsequent blocking of endogenous peroxidase by treatment with Peroxidase-Blocking Solution (S2023; Dako) for 7 min, followed by incubation with primary antibodies at room temperature for 25 min. Next, slides were re-washed in buffer, and treated with the secondary antibody, EnVision Flex, High pH (Link) (K800; goat anti-mouse/human polyclonal; Dako) for Ki-67 and S-100 and Real EnVision (K5007; horseradish peroxidase; goat anti-mouse/rabbit polyclonal; DAKO). After 25 min of incubation and two further rinses with buffer for CD99, slides were covered with the visualization agent, DAB (K3468; Dako) for 10 min, rinsed, counterstained in EnVision Flex Haematoxylin and mounted. All cells that showed immunoreactivity for S-100 in the cartilage and CD99 in the spindle cell component were counted at high power fields (magnification, ×400) using a Nikon Eclipse E800 microscope (Nikon Corporation, Tokyo, Japan).

### RNA extraction, reverse transcription-polymerase chain reaction (RT-PCR) and sequencing

Paraffin-embedded tumour tissue was histologically analysed, and a representative block was selected for the presence of viable tumour cells selected. Thirty 5-μm sections were used for total RNA extraction, using the RNeasy FFPE kit (Qiagen, Hilden, Germany). The concentration of extracted total RNA was determined with a NanoDrop 2000 spectrophotometer (Thermo Scientific, Waltham, MA, USA).

An aliquot of 300 ng total RNA was reverse-transcribed and amplified using the Qiagen OneStep RT-PCR kit (Qiagen). RT-PCR was conducted using the following primer set: HEY1 forward (exon 4), 5′-ACCGTGGATCACCTGAAAAT-3′ and NCOA2 reverse (exon 13), 5′-TGCAATGTGATGTCAAGTGG-3′, at an annealing temperature of 61°C and for 40 cycles. The primers amplified a 119-bp product representing a fragment of HEY1 exon 4 fused in-frame to NCOA2 exon 13 was amplified. As a positive control for the RNA integrity, RT-PCR for the housekeeping gene, β-actin, was performed using the following primer set: β-actin forward, 5′-ATCACCATTGGCAATGAGCG-3′ and reverse, 5′-TTGAAGGTAGTTTCGTGGAT-3′, at an annealing temperature of 61°C and for 40 cycles. These primers amplified a 100-bp fragment of the housekeeping gene β-actin. An aliquot of the amplified RT-PCR products was visualised by electrophoresis on a 2% agarose gel stained with ethidium bromide.

The amplified RT-PCR product was purified using Illustra Microspin S-300 HR columns (GE Healthcare, Ltd., Chalfont St. Giles, United Kingdom). To confirm the presence of the fusion transcript, the purified product was sequenced using the BigDye Terminator v1.1 Cycle Sequencing kit and resolved on a 3130xl Genetic Analyzer (Applied Biosystems, Foster City, CA, USA). Subsequently, sequences were manually aligned with the original sequences of HEY1 (NM_012258.3) and NCOA2 (NM_006540.2) for comparison.

### Histological and immunohistochemical results

**Figure 1:**
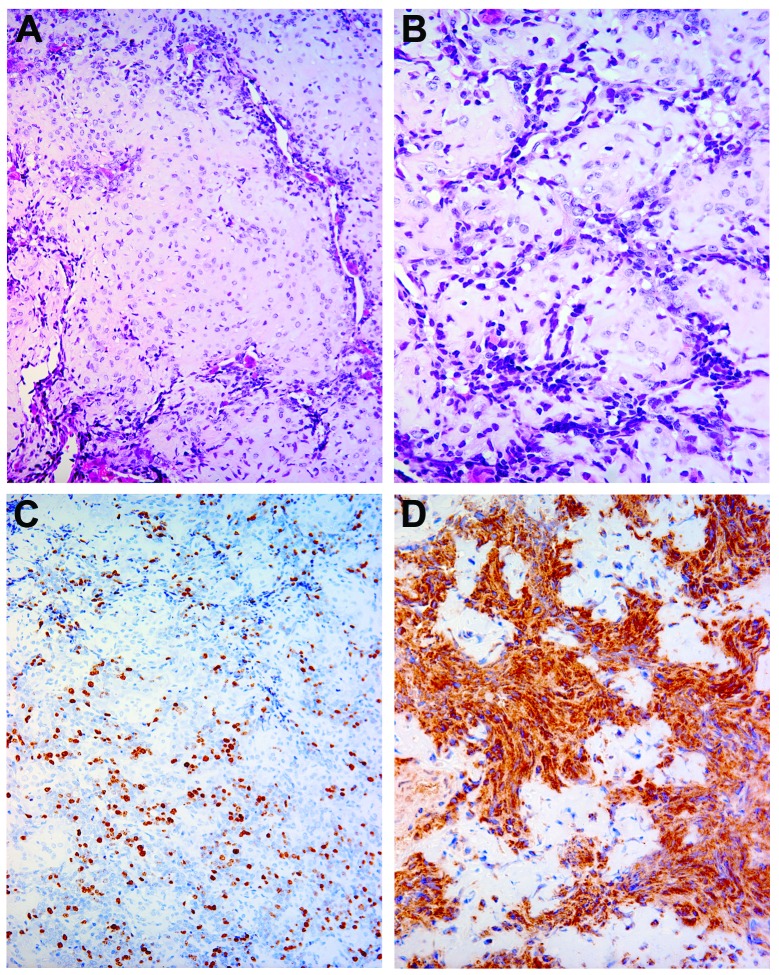
(A and B) Typical morphological features of mesenchymal chondrosarcomas showing a biphasic pattern of cartilage islands distributed among spindle cells, mainly located in the periphery. Chondrocytes showed moderate nuclear atypia, while spindle cells exhibited nuclear hyperchromatism and pleomorphism [staining, haematoxylin and eosin; magnification, ×200 (A) and ×400 (B)]. (C) The proliferation index (Ki-67) was high in the spindle cell component, but low in the cartilage islands (magnification, ×200). (D) After staining for CD99 (MIC 2), strong immunoreactivity was observed only in the peripheral cellular part of the tumour (magnification, ×400).

Macroscopically, the tumour was chondromatous with bone fragments displaying a grey-white and pink colour with soft-to-firm consistency. No necrosis or haemorrhage was observed. Microscopically, the tumour showed a biphenotypical appearance, with both hypercellular and hypocellular areas. In terms of cellular components, the tumour showed undifferentiated small round-to-ovoid shaped cells with brisk mitotic activity and hyperchromasia. Hypocellular islands with chondroid tissue and well-differentiated hyaline cartilage were observed (Fig. 1A and B). Immunohistochemical analysis disclosed that all undifferentiated cells stained positive for CD99 (Fig. 1D), with a high degree of positivity for Ki-67 (25%) (Fig. 1C) and for S-100 (100%) in the cartilage component (data not shown).

### RT-PCR results

**Figure 2:**
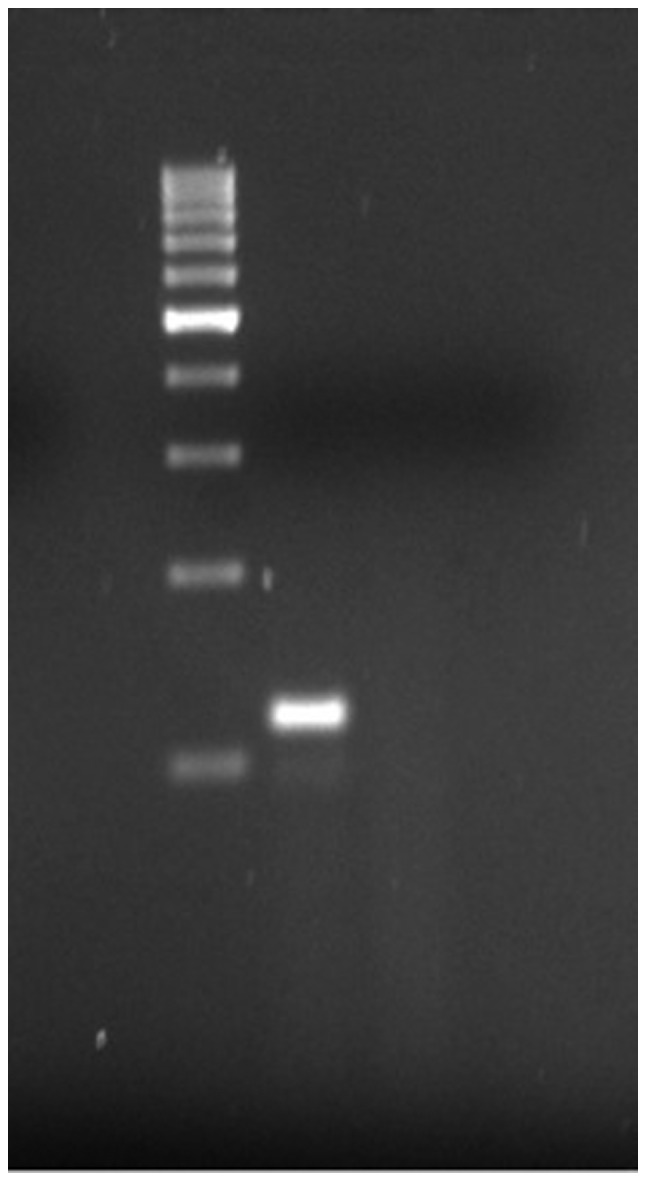
RT-PCR results. Paraffin-embedded tumour tissue was used for the RT-PCR. RT-PCR with specific primers for HEY1-NCOA2 fusion gene showed a strong band of 119 bp. RT-PCR, reverse transcription-polymerase chain reaction.

**Figure 3:**
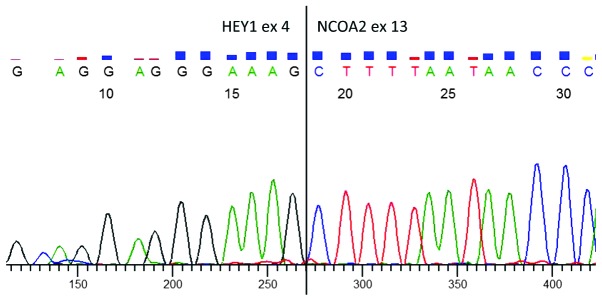
Primers were derived from exon 4 of HEY1 and exon 13 of NCOA2. Following direct sequencing of the reverse transcription-polymerase chain reaction product, sequences were manually aligned with the original sequences of HEY1 (NM_012258.3) and NCOA2 (NM_006540.2).

RT-PCR for the HEY1-NCOA2 fusion gene was attempted, as paraffin-embedded tumour material was available. A strong 119-bp band was obtained (Fig. 2) upon amplification using a forward primer derived from exon 4 of HEY1 and reverse primer from exon 13 of NCOA2. Direct sequencing of the product confirmed the presence of a HEY1-NCOA2 chimeric transcript with a junction between HEY1 exon 4 and NCOA2 exon 13 (Figs. 2 and 3).

## Discussion

In the current study, the HEY1-NCOA2 fusion gene was detected in a paediatric case of intraspinal MCS. These findings validate those of a recently published primary report regarding this gene fusion in MCS at an intraspinal location (19).

MCS, as observed in the current case, is an extremely rare tumour that is morphologically characterized by a biphasic pattern of small, round, undifferentiated hyperchromatic cells with islands of cartilage with a varying degree of hyaline differentiation. Approximately 70% of all MCS cases occur in the bone, with the remaining detected in extraskeletal locations, as observed in this case. In contrast to classical chondrosarcomas, MCS is an aggressive, fast-growing tumour that frequently metastasises and has the ability to remain dormant for long periods of time. Furthermore, as seen in this case, MCS tends to affect children and young adults, in contrast to classical chondrosarcomas that mostly affect patients over 50 years of age. MCS accounts for <1% of all sarcomas (20) and represents only 2–10% of all chondrosarcomas (2). The tumour preferentially metastasises to the lung, lymph nodes and other bones (6,7). To date, with the present case included, only 15 cases of intraspinal (meningeal) MCS have been published (6,7,21–32), and seven of these occurred in children (7,21,24,27,30–32). These tumours arise most frequently in the mid-level of the spine, specifically, the lower thoracic and upper lumbar region; although, in certain cases, tumours have been identified in the cervical spine (26,33) as well as the sacral part of the spine (22).

As expected, symptoms associated with these tumours reflect their compressive effects on the specific neuroanatomical structure. As was the condition of the present case, the clinical findings of MCS are often subtle and non-specific. Typically the patient presents with focal back pain, stiffness and, on occasion, sensory-motor signs of spinal cord compression, such as weakness. The duration of symptoms also differs considerably, ranging from weeks to many years, often leading to late investigation and diagnosis. However, in the majority of reported cases, the initial symptom is pain, probably as a consequence of local swelling, similar to the current report (1,34).

In accordance with the standard investigative procedures of bone tumours, clinical, radiological and pathological examinations are necessary to obtain a correct diagnosis. Radiological findings of MCS typically comprise an osteolytic diffusely demarcated lesion with punctate calcifications. On plain radiographs, these lesions exhibit radiolucent areas with matrix calcification, such as arcs and rings (1,7,35). However, no specific MRI findings to distinguish mesenchymal chondrosarcomas from ordinary types of chondrosarcomas have been established thus far.

The rarity of the tumour, in combination with different origins, such as bone, soft tissue, brain, meninges and spinal tissue (25,33,36), prevents conclusive studies. Consequently, the underlying tumour mechanisms are therefore poorly understood (2), and there is no general agreement with regard to the best choice of therapy (3). Primary treatment is based on surgery, although some centres have used adjuvant radiotherapy as well as chemotherapy (9). The effectiveness of adjuvant chemo- and/or radiotherapy in conjunction with surgery is not well-defined (5,20,37–39). Unsurprisingly, it is concluded that wide surgical resection margins improve the survival outcome (10). Earlier studies have additionally suggested that survival is affected negatively and positively by specific factors, such as proliferation rate (12) and tumour origin in bone (10), respectively.

The HEY1-NCOA2 gene fusion detected in the present study has, to date, only been detected in MCS, but has been absent in all types of chondrosarcoma that have been investigated (19). This finding is of clinical as well as scientific value, since identification of a specific molecular marker should effectively distinguish MCS from other types of morphologically similar sarcomas, and further provide a key to the resolution of pathogenesis, since NCOA2 interacts with specific ligand-bound nuclear receptors that facilitate chromatin remodelling and transcription of nuclear receptor target genes (40). NCOA2 is included in the nuclear receptor transcriptional co activator family (41). Notably, NCOA2 has been detected as a fusion partner in numerous other malignancies, including acute myeloid leukaemia (MYST3-NCOA2) (42), other types of acute leukaemia (ETV6-NCOA2) (43), subtypes of alveolar rhabdomyosarcoma (PAX3-NCOA2) (44) and, recently, benign soft tissue angiofibromas (AHHR-NCOA2) (45). These examples demonstrate that despite divergent clinical behaviour and outcomes between different diagnoses, the tumours commonly contain NCOA2 as a fusion partner. Further studies are required to provide evidence of how these genes are involved in the pathogenesis of neoplastic disorders. Furthermore, the mechanisms underlying the high specificity of the HEY1-NCOA2 gene fusion for MCS and its potential utility in the development of new therapeutic approaches remain to be established.
